# Evaluation of bone mineral density of the lumbar spine using a novel phantomless dual-energy CT post-processing algorithm in comparison with dual-energy X-ray absorptiometry

**DOI:** 10.1186/s41747-017-0017-2

**Published:** 2017-09-20

**Authors:** Christian Booz, Philipp C. Hofmann, Martin Sedlmair, Thomas G. Flohr, Bernhard Schmidt, Tommaso D’Angelo, Simon S. Martin, Lukas Lenga, Doris Leithner, Thomas J. Vogl, Julian L. Wichmann

**Affiliations:** 1 Department of Diagnostic and Interventional Radiology, University Hospital Frankfurt, Theodor-Stern-Kai 7, 60590 Frankfurt, Germany; 2 Siemens Healthcare, Computed Tomography Division, Forchheim, Germany; 3 Department of Biomedical Sciences and Morphological and Functional Imaging, University of Messina, Messina, Italy

**Keywords:** Bone mineral density, Osteoporosis, Dual-energy X-ray absorptiometry, Dual-energy computed tomography, Computed tomography

## Abstract

**Background:**

Current techniques for evaluation of bone mineral density (BMD) commonly require phantom calibration. The purpose of this study was to evaluate a novel algorithm for phantomless in vivo dual-energy computed tomography (DECT)-based assessment of BMD of the lumbar spine in comparison with dual-energy X-ray absorptiometry (DEXA).

**Methods:**

Data from clinically indicated DECT and DEXA examinations within two months comprising the lumbar spine of 47 patients were retrospectively evaluated. By using a novel automated dedicated post-processing algorithm for DECT, the trabecular bone of lumbar vertebrae L1–L4 was selected and analysed. Linear correlation was analysed using Pearson’s product-moment correlation coefficient for the comparison of the results from DECT and DEXA.

**Results:**

A total of 186 lumbar vertebrae in 47 patients (mean age, 58 years; age range, 24–85 years) were analysed, 24 men (mean age, 55 years; age range, 24–85 years) and 23 women (mean age, 59 years; age range, 31–80 years). Mean BMD of L1–L4 determined with DEXA was 0.985 g/cm^2^ and 20/47 patients (42.6%) showed an osteoporotic BMD (T score lower than – 2.5) of at least two vertebrae. Average DECT-based BMD of L1–L4 was 86.8 mg/cm^3^. Regression analysis demonstrated a lack of correlation between DECT- and DEXA-based BMD values with a Pearson’s product-moment correlation coefficient r = 0.4205.

**Conclusions:**

Dedicated post-processing of DECT data using a novel algorithm for retrospective phantomless BMD assessment of the trabecular bone of lumbar vertebrae from clinically indicated DECT examinations is feasible.

## Key points

- Phantomless DECT-based bone mineral density (BMD) assessment is feasible.
- DECT provides volumetric BMD assessment.
- DECT-based volumetric BMD assessment showed lack of correlation with areal DEXA (r = 0.4205).
- Simultaneous phantomless BMD evaluation during diagnostic CT may reduce cumulative radiation dose.

## Background

Osteoporosis is a common metabolic bone disorder, especially in the elderly population, which is characterised by a loss of bone mineral density (BMD) and an alteration of bony microarchitecture [1, 2]. It is associated with a drastically increased risk of fractures of the hip, spine and wrist, deformations of bone and consecutive malpositioning of the skeletal system [3, 4].

According to the official positions of the World Health Organization (WHO) [1], the gold standard for diagnosis and assessment of osteoporosis is the evaluation of BMD by using dual-energy X-ray absorptiometry (DEXA). The advantages of DEXA are its relatively low cost, non-invasiveness and low radiation exposure for patients. However, multiple studies have also demonstrated certain limitations of DEXA such as distortion of values estimating actual bone mass and interference by body composition [5–7]. There are also potential errors of DEXA due to common pitfalls consisting of patient positioning, acquisition, data analysis and artefacts that may impair DEXA results [8]. While DEXA can provide an accurate bone density measurement in vitro [9], in vivo analysis is impaired by overlying soft tissue, vascular calcifications, bowel contents and degenerative spine changes [7, 10]. In addition, DEXA, as a two-dimensional (2D) scanning examination method, measures an areal density (g/cm^2^) of the whole vertebral body. However, the inner trabecular bone has been shown to be a metabolically more active tissue compared with the outer cortical bone and is therefore more influenced by changes in bone mass [11]. A three-dimensional (3D) imaging procedure confined to the trabecular bone would allow a more detailed assessment of changes in BMD [12–15].

Dual-energy computed tomography (DECT) is an imaging technique which has been used for quantitative imaging due to its ability for material differentiation [12, 13]. First studies involving early DECT concepts for BMD evaluation were published more than two decades ago [12, 13]. A novel approach for regional in vitro BMD assessment with DECT was presented in 2012 [14]. In this study, Wesarg et al. demonstrated that DECT allows the assessment and 3D display of spatial BMD distribution, facilitating a more detailed evaluation of focal bone solidity compared with DEXA [14]. Wichmann et al. consequently performed a study using that algorithm, in which they could show that phantomless in vivo DECT-based BMD assessment of the lumbar spine in a clinical setting is feasible [15]. While the algorithm evaluated in these studies was available as stand-alone software, it could not be integrated into standard post-processing software offered by vendors. Thus, export of DECT datasets and analysis on a separate machine was necessary. In this current study, we evaluated a novel prototype algorithm for phantomless 3D evaluation of BMD developed directly by a vendor which can be integrated into its offered DECT postprocessing system to allow the automated assessment of BMD within common post-scan clinical workflows. The goal of our study was to evaluate this novel algorithm regarding its ability for 3D in vivo DECT-based phantomless assessment of volumetric BMD of the lumbar spine in comparison with 2D DEXA.

## Methods

### Patient selection and study design

This retrospective study was approved by the institutional review board and the requirement to obtain informed consent was waived.

We retrospectively included patients that had undergone both a DECT examination that comprised the lumbar spine and a DEXA examination between April 2012 and August 2014. To limit possible distortion of the statistical correlation between DECT and DEXA, we only included data from patients with an interval of up to two months (60 days) between the two examinations. DEXA for BMD measurement was clinically indicated for diagnosis of osteopenia or osteoporosis. Diagnostic DECT of the abdomen, pelvis or spine was performed to rule out fractures in patients with known osteoporosis (n = 16), to evaluate spinal structures and rule out fractures in patients with lumbago (n = 9), to rule out malignancy (n = 8) or to restage tumours in patients with known lymphoma (n = 6). We excluded patients with diffuse skeletal metastases (n = 3), multiple myeloma (n = 4), as well as patients aged less than 18 years. Further exclusion criteria were lumbar vertebrae of patients with metallic implements after spinal surgery or hip replacements due to possible beam-hardening artifacts (n = 6), presence of malignancy of the spine or adjacent to the spine (n = 4), lumbar vertebrae showing signs of vertebral compression fracture or other types of fractures (n = 10). Detailed patient characteristics are summarised in Table 1.

**Table 1:** Characterisation of the patient population in this study (n = 47)

Characteristics	Value
Age ± SD, years (range)	58.05 ± 13.0 (24–85)
Male, n (%)	24 (51)
BMI ± SD, kg/m^2^ (range)	24.57 ± 3.72 (16.73–35.23)
Known diseases affecting BMD, n (%)	
Osteoporosis	16 (34)
Cystic fibrosis	5 (10.6)
Malignancy	30 (64)
Renal failure	12 (25.5)
Stem cell transplantation	8 (17)
Known medications affecting BMD, n (%)	
Cholecalciferol	24 (51)
Calcitriol	9 (19)
Corticosteroids	13 (27.7)
Bisphosphonates	13 (27.7)

*SD* standard deviation, *BMD* body mass density

### DEXA scan protocol

DEXA was performed by using standard techniques according to manufacturer and WHO guidelines [1]. A Lunar Prodigy Advance bone densitometer (GE Healthcare, Madison, WI, USA) was used. Images of the lumbar spine (L1–L4) were obtained in posterior-anterior acquisition. For each vertebra, the manufacturer software automatically calculated BMD values and standardised T scores and Z scores on the basis of age- and sex-matched control participants.

According to WHO guidelines [1], a DEXA-derived T score < 1.0 indicates an abnormally low BMD, which is further categorised into osteopenia (T score between – 1.0 and – 2.4) and osteoporosis (T score of ≤ 2.5).

Similar to routine clinical practice, the diagnosis of osteopenia or osteoporosis was based on the lowest measured central T score of at least two evaluated lumbar vertebrae while all lumbar vertebrae L1–L4 were analysed. Only DEXA results of the lumbar spine were included, as results of hip DEXA were available only in a fraction of the patients.

### DECT scan protocol

The CT examinations in our study were performed by using a second-generation 128-section dual-source CT system in dual-energy mode (Somatom Definition Flash; Siemens Healthcare, Forchheim, Germany). The two X-ray tubes were operated at different kilovoltage settings (tube A = 140 kVp with a tin filter and 105 mAs per rotation; tube B = 80 kVp with 165 mAs per rotation). Other scanning parameters were rotation time of 280 ms and a pitch of 0.17. A collimation of 2 × 64 × 0.6 mm with z-flying focal spot technique was used with both detector systems. Image series were acquired in the craniocaudal direction with patients in a supine position and both arms extended above the head. When patients had been referred for an abdominal examination, the anatomic range extended from right above the diaphragm to just below the sacrum. If patients had been referred for a CT examination of the lumbar spine, the anatomic range extended from vertebra T12 to just below the sacrum.

Images were reconstructed with a dedicated dual-energy bone kernel (D70f) and the recorded information of the full gantry rotation (temporal resolution of 280 ms) with a section thickness of 1.5 mm and an increment of 1.0 mm.

### Post-processing of DECT data

**Fig. 1:**
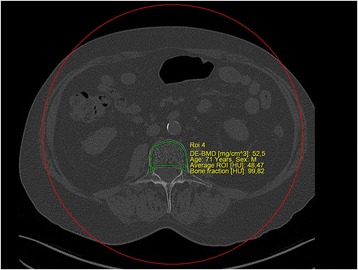
After automatic placement by the post-processing software (Examine, Siemens), the VOI was manually defined by the user in order to achieve the best delineation of the trabecular bone and exclusion of any cortical bone. Five VOIs were placed for each vertebra

The software used for computation of the trabecular bone in our study (Examine, Siemens Healthcare) required prior delineation of the volume of interest (VOI). To achieve this, the VOI was manually defined by the user in order to achieve the best delineation of the trabecular bone and exclusion of any cortical bone (Fig. 1). Five VOIs on different slices were manually defined for each vertebral body. This was repeated throughout the whole stack of 2D slices for every vertebra to be included in the analysis. The labelled volumes served as input for the analysis software together with the two image datasets representing the low-energy and the high-energy DECT scans. The post-processing algorithm was based on the following steps. First, the VOI was manually defined. Second, an internal beam-hardening correction for the VOI was carried out by the software. A three-material decomposition (bone, red bone marrow, yellow bone marrow) was performed for each voxel. The VOIs of the low-energy and high-energy levels were analysed by the software using specific mathematical algorithms to calculate the energy level-dependent absorption of radiation of each voxel. Based on the absorption, the Hounsfield units (HU) were calculated. Finally, HU were transformed into milligrams hydroxyapatite per cubic centimetre (mg/cc) representing the BMD. Labelling and BMD analysis were performed on a commercially available personal computer (ThinkPad®Lenovo R61, IBM, Armonk, NY, USA).

### Statistical analysis

Statistical analysis was performed using commercially available statistical software (IBM SPSS Statistics, version 21, IBM; MedCalc Statistical Software Version 16.4.1, MedCalc Software bvba, Ostende, Belgium). Evaluation of normality of the data was performed using the Shapiro–Wilk test. Variables were expressed as median or mean ± standard deviation. Linear correlation was analysed using Pearson’s product-moment correlation coefficient for the comparison of results from DECT with DEXA.

## Results

A total of 207 lumbar vertebrae in 47 patients were examined by DEXA and DECT in this study. Twenty-one lumbar vertebrae were not completely examined and therefore excluded. The remaining 186 lumbar vertebrae were included and analysed. Mean patient age was 58.05 ± 13.0 years (age range, 24–85 years). Twenty-four patients (51%) were men (mean age, 54.8 years; age range, 24–85 years) and 23 patients were women (mean age, 59.4 years; age range, 31–83 years). Average body mass index was 24.57 ± 3.72 kg/m^2^ (range, 16.73–35.23 kg/m^2^).

DEXA-derived calculated average bone density of L1–L4 was 0.985 ± 0.306 g/cm^2^ (range, 0.661–1.420 g/cm^2^). According to WHO guidelines, DEXA measurements of at least two vertebrae or more identified ten patients (21%) with an osteopenic BMD with a T-score between – 1.0 and – 2.5. Twenty patients (42.6%) showed an osteoporotic BMD with a T-score of – 2.5 or below measured by DEXA of at least two vertebrae.

All lumbar vertebrae could be analysed by using the dedicated software for BMD computation. The overall mean DECT-based BMD value of L1–L4 was 86.8 mg/cm^3^ ± 33.5 mg/cm^3^ (range, 28.5–289.1 mg/cm^3^). According to American College of Radiology (ACR) guidelines, DECT measurements identified 17 patients (36%) with an osteoporotic BMD. The results of BMD assessment based on the used approaches are summarised in Table 2.

**Table 2:** The results of BMD assessment using DEXA and DECT

	DXA	DECT
Patients (n)	47	47
Lumbar vertebrae (n)	186	186
Mean BMD	0.985 ± 0.306 g/cm^2^	86.8 ± 33.5 mg/cm^3^
Range	0.661–1.420 g/cm^2^	28.5–289.1 mg/cm^3^
Diagnosis of osteoporosis (n)	10^a^	17^b^

*BMD* body mass density, *DEXA* dual energy X-ray absorptiometry, *DECT* duale energy computed tomography

^a^According to WHO guidelines [1]

^b^According to ACR guidelines [18]

Regression analysis demonstrated a lack of correlation between DECT- and DEXA-based BMD values with a Pearson’s product-moment correlation coefficient r = 0.4205 (*p* < 0.0001).

## Discussion

In this retrospective study, we demonstrated that phantomless 3D DECT-based in vivo BMD assessment of standard DECT datasets using a novel vendor-specific algorithm is feasible. Similar to other studies, we found no statistical correlation between DEXA and DECT values, which can be expected since DECT measures true volumetric BMD confined to the trabecular bone and DEXA measures areal BMD referring to both cortical and trabecular bone [14, 15]. While prior studies on DECT-based BMD assessment of the lumbar spine employed stand-alone software which required data export and could not be integrated into clinical workflows, the novel algorithm analysed in our study was specifically developed by the vendor and will therefore become available within its dedicated post-processing software suite in the future (syngo.via, Siemens). Similar to other applications of quantitative DECT (e.g. imaging of gout, gall stone characterisation), the purpose of this algorithm is to provide additional information from routinely performed DECT examinations without any additional radiation exposure. Nevertheless, specific BMD reference values for the diagnosis of osteoporosis using DECT have to be developed in future larger studies to make use of this technique in clinical routine.

Pickhardt et al. were the first to demonstrate that phantomless BMD evaluation of the lumbar spine is feasible during CT colonography [16]. They reported that a threshold of 0.09 g/cm^3^ at quantitative computed tomography (QCT) yielded 100% sensitivity (29/29) for the detection of osteoporosis with a specificity of 63.8% (143/224) in comparison with DEXA results. When analysing attenuation measurements of lumbar vertebrae, they found that a threshold of 160 HU was 100% sensitive for osteoporosis with a specificity of 46.4%. In another recently published study, Budoff et al. investigated phantomless BMD measurements of the thoracic spine during coronary CT angiography [17]. In their study, phantomless BMD assessment correlated highly with phantom-based QCT of the thoracic spine although the calibration factors differed substantially within each CT scanner model. It should be noted that in both these studies standard QCT phantoms were initially used to develop conversion factors which we did not employ in our study. In addition, the approach for phantomless CT-based BMD assessment in the aforementioned and other studies has been mostly based on evaluation of attenuation measurements using manually drawn ROIs. While there is certainly a correlation between decreased BMD and lower HU values, we do believe that this approach may play a role as a simplified screening test, but does not make use of the quantitative data which can be obtained using DECT to obtain a more specific diagnosis.

Nevertheless, the results of our and prior studies emphasise the known issues regarding the correlation of measurements derived from quantitative CT imaging in general and DEXA. We plan to evaluate larger patient cohorts to correlate DECT-, QCT- and DEXA-based BMD results to calculate a conversion factor for the QCT guidelines published by the ACR [18].

Patients with prolonged drug treatment, chronic diseases such as cystic fibrosis, after organ transplantation or cancer survivors commonly undergo follow-up DEXA-based BMD assessment in addition to repeated diagnostic CT scans [19, 20]. Younger patients in particular may benefit from automatically obtained BMD measurements during regularly performed diagnostic DECT examinations to assess changes in trabecular BMD and allow for early detection of osteoporosis.

There are certain limitations to our study which warrant discussion. First, as we focused on demonstrating that DECT-based assessment of BMD is feasible in a clinical setting and the interval between DECT and DEXA was limited to 60 days to avoid distortion of results, only 47 patients were included in this initial study. While in vitro measurements were performed by the vendor during the development of this algorithm, no in vitro experiments were performed in the context of our study. Thus, additional phantom studies evaluating the reproducibility of BMD measurements using this algorithm are necessary. Second, the clinical indications for CT imaging may have led to potential selection bias. A multicentre approach with a larger patient cohort and comparison of subgroups with more homogeneous diseases is required to reassess the practicability of this technique in routine clinical practice. Third, this novel algorithm was developed by a single vendor for inclusion into its dedicated post-processing software suite. Thus, this algorithm and our results do not apply to DECT realisations from other vendors. Fourth, outcome data were not available within the context of this feasibility study. Thus, the ability of DECT-based BMD assessment to predict osteoporosis-related complications, such as vertebral compression fractures, remains unclear. Fifth, the prevalence of osteoporosis is known to be higher among women [21]. In our retrospective study, 51% of the patient population were men. This could have influenced our results regarding the presence and degree of osteoporosis. Sixth, only one operator performed VOI measurements leading to no inter-operator analysis. However, similar to this approach, DEXA results are also dependent on the operator’s experience and confinement of VOIs but are not repeated in clinical practice. In addition, the next development steps for this algorithm include automated detection and delineation of trabecular bone. Thus, no manual VOI measurements would be performed once these algorithms have been included and tested.

## Conclusions

In conclusion, we demonstrated that phantomless in vivo DECT-based BMD assessment of the lumbar spine in a clinical setting using a novel algorithm directly developed by a vendor is feasible. Further studies with larger patient groups are necessary to establish reference values for DECT-derived BMD values for the diagnosis of osteoporosis.

## Abbreviations

ACR: American College of Radiology
BMD: Bone mineral density
DECT: Dual-energy computed tomography
DEXA: Dual-energy X-ray absorptiometry
HU: Hounsfield unit
QCT: Quantitative computed tomography
ROI: Region of interest
VOI: Volume of interest
WHO: World Health Organization
